# Congruency of gaze metrics in action, imagery and action observation

**DOI:** 10.3389/fnhum.2013.00604

**Published:** 2013-09-24

**Authors:** Joe Causer, Sheree A. McCormick, Paul S. Holmes

**Affiliations:** ^1^Brain and Behaviour Laboratory, Liverpool John Moores UniversityLiverpool, UK; ^2^Centre for Cognitive Motor Function, Institute for Performance Research, Manchester Metropolitan UniversityCrewe, UK

**Keywords:** action observation, congruency, eye movements, motor learning, movement imagery, neuroscience

## Abstract

The aim of this paper is to provide a review of eye movements during action execution, action observation, and movement imagery. Furthermore, the paper highlights aspects of congruency in gaze metrics between these states. The implications of the imagery, observation, and action gaze congruency are discussed in terms of motor learning and rehabilitation. Future research directions are outlined in order to further the understanding of shared gaze metrics between overt and covert states. Suggestions are made for how researchers and practitioners can structure action observation and movement imagery interventions to maximize (re)learning.

Neuroimaging techniques, such as functional magnetic resonance imaging (fMRI), have allowed researchers to locate specific areas of brain activation and highlight the spatial and temporal congruency between observing, executing, and imaging actions. There is now a common understanding that the covert elements (attention, motor planning) of action execution, action observation and movement imagery share, at least in part, similar neural networks and mechanisms (Grézes and Decety, 2001; Holmes et al., 2010). For example, activation of motor cortex and ventral parts of pre-motor cortex has been reported during observation of conspecific actions (Fadiga et al., 1995), as well as movement imagery of an action (Gerardin et al., 2000). Despite significant evidence proposing a partially shared neural pathway, there remains a distinct lack of research identifying the processes by which individuals use information in each of these states and whether there is meaningful congruency between the states. In contrast to imaging techniques such as fMRI, one method of quantifying imagery and observation of goal-directed action is by measuring eye movements, which may provide an online indication of some of the attentional and cognitive processes (Liversedge and Findlay, 2000). This may inform the debate on the meaningfulness of any shared neural substrate. This paper therefore, provides a review of eye movements during action execution, movement imagery and action observation and highlights aspects of congruency in gaze metrics between these states. For a range of gaze metrics we consider clinical and research implications, and translational applications across a number of domains and provide several key research areas that we propose would benefit from further inquiry.

## Gaze in action execution

An extensive body of research suggests that vision is the dominant sensory system underpinning human function (Causer et al., 2012) and the processes and mechanisms by which vision aids and controls movement have been researched extensively (Elliott et al., 2012). During perception, external visual information is retinoptically mapped (preserved) onto topographically organized areas in the occipital lobe. The “attended” environmental visual cues are then processed via the dorsal, ventral, and rostral streams of the visual system; the dorsal stream permitting identification of object location, size and orientation, the ventral stream facilitating object recognition, and the rostral stream acting as a conduit between both (Goodale and Milner, 1992). In the dorsal stream, which extends into the posterior parietal cortex, the visual and other sensory information is transformed into a common eye-centered frame of reference in motor areas to guide movement (Andersen et al., 1997; Desmurget et al., 1999). Although the degree of correspondence between gaze and stimulus may vary depending upon nature of the task (Frens and Erkelens, 1991; Binsted and Elliott, 1999), the majority of everyday actions such as reaching for a cup or catching a ball are considerably easier and often more accurate with vision. Typically, specific eye movements (visual fixations) precede motor manipulation (Abrams et al., 1990) and during visuomotor tasks such as reach and grasp, the location and duration of these unique eye movements are considered to perform two vital monitoring functions: (1) identifying the goal directed target; and (2) providing visual feedback about the grasping hand to enable online corrections (Land et al., 1999; Brouwer et al., 2009).

Seminal work by Woodworth (1899) suggested that once a stationary target is identified a single ballistic movement occurs that brings the limb into the vicinity of the target. This is then followed by a single corrective movement that is based on visual feedback about the relative positions of the limb and target. Woodworth suggested that the corrective part of the movement involved a graded “homing” in on the target. Over a century later the basic tenets of the two-component theory are still supported by researchers examining the active control of goal-directed movements.

## Gaze in action observation

When teaching a movement or skill, demonstrations are frequently used by the instructor (Magill, 2000). These demonstrations are argued to modify behavior through by various mechanisms. For example, an individual may adapt their behaviors to match a model (echokinesis: Prinz, 1987; imitation: Heyes, 2001), an object (emulation: Heyes, 2001) or a perceived goal intention or outcome (Byrne and Russon, 1998). In the skill acquisition/motor learning literature however, observational learning, often referred to as modeling, is seen as more pertinent. Observational learning can be defined as the process by which an individual observes a behavior and adapts his/her action(s) accordingly (Bandura, 1986). The critical difference between observational learning and imitation or emulation is the focus on long-term learning of a skill and a relatively permanent change in behavior rather than a discrete performance. Learning by observation, as well as the ability to recognize and interpret the movements, actions, and goals of others all rely on action observation. Below we will discuss how eye movements are utilized during action observation and the similarities between observing and executing actions.

The direct matching hypothesis (Flanagan and Johansson, 2003) postulates that observing behaviors performed by others elicits motor activity in the brain of the observer similar to that which occurs when the individual plans his/her own actions (Rizzolatti et al., 2001; Rizzolatti and Sinigaglia, 2010). Transcranial magnetic stimulation (TMS) studies have demonstrated that during the observation of goal-directed movements, an increase in a muscle specific motor evoked potential occur in the human motor cortex (Fadiga et al., 1995), and that predictive eye movements are linked to the invoked motor program (Elsner et al., 2013). When observing someone else acting on an object, people implement goal-specific eye movement programs that are driven by their own motor representation for the transient action. Falck-Ytter et al. (2006), for example, demonstrated that proactive goal-directed eye movements in adults result from the direct matching of an observed action with the motor representation of that action. Further, they showed that infants gaze proactively toward the target object of others' actions at the same age as they become able to perform those actions themselves. Elsner et al. (2013) also found that during observation of a conspecific reaching to a target object, stimulation of the observer's motor cortex disrupted the ability to predict the observed actions and was also indexed by delayed predictive eye movements demonstrating eye gaze coupling with motor output.

Action observation is also influenced by observation strategy instructions associated with the stimuli observed. For example, Decety (1996) reported that the neural profile was altered depending on whether the task was to “recognize” the action or to “observe the action with the intent to imitate.” Only in the “intent to imitate” condition were areas involved in the planning and generation of movement activated. In addition, the activation was also differentiated by the stimuli presented. Individually-meaningful actions activated the left frontal and temporal (planning) areas whilst meaningless actions activated the right occipital-parietal area. Eye movements have also been shown to be influenced by task strategy. Brouwer et al. (2009) demonstrated a different eye movement pattern dependent upon whether the action involved the viewing of a stationary object or the reach and grasp of that object. During viewing, the eyes fixated the center of mass of the object, whilst during reach and grasp the eyes predictively fixated the future contact areas of the index finger and thumb. These data suggest that when motor plans are generated, gaze performs an active role in action observation, linked to sensory prediction, just as it does in action execution and should be considered in research protocols and intervention designs when providing instructions to participants.

## Gaze in movement imagery

Imagery has been shown to influence motor processes, such as the kinematics, kinetics and co-ordination of action, and cognitive process such as motivation, attention and affect (Holmes and Collins, 2001). The use of imagery and, in particular, movement imagery, defined as the representation of human action in the absence of movement execution (Jeannerod and Frak, 1999), has practical implications in a range of domains: music; sport; surgery; military settings; and clinical rehabilitation. Practicing movement imagery, either discretely or, better, in conjunction with physical practice, has been reported to improve motor skill acquisition and performance (Page et al., 2005; Dickstein and Deutsch, 2007).

The concept of eye movement metrics as a useful marker of imagery behavior is not new and the role of gaze and eye movement congruence in imagery has been known for some time. It is surprising, therefore, that researchers and practitioners have not considered the importance of eye movements for image generation. For example, Hebb (1968) suggested that if an image is a reinstatement of the perceptual process then it should include similar eye movements and be constructed in a similar manner. During imagery, the object recognition system (occipital areas and ventral stream) is thought to be primed strongly causing a pattern of neural reactivation (the visual image) to be generated (Kosslyn, 1995). The iterative retrieval of information in the reconstruction of the image is suggested to be assisted by an occulomotor-based coordinate system; eye fixations during perception are encoded, stored alongside the visual representation and used later as an index during systematic image generation (Laeng and Teodorescua, 2002). This suggests that congruent eye movement metrics are an important component in image generation and contribute to two key aspects of the image; its control and quality. This concept is similar to that suggested to operate during visually guided action execution; the action is planned, and updated, in common eye-centered coordinates using information from sensory stimuli and motor effectors (Batista et al., 1999). As with perception, it is possible to scan the visual image and direct attention to key features thus permitting the image's complexity and vividness to be “built up” over time (Kosslyn, 2010). If imagery can be used during skill acquisition and motor (re)learning and eye movements perform a functionally meaningful role, then the efficacy of imagery as a technique for (re)learning may be greater if the eye movements are monitored and controlled during the imagery process.

Despite the extensive research into imagery and imagery mechanisms there remains a paucity of research examining eye movements in movement imagery, where the visual component is clearly important (Jeannerod, 1994). Rodionov et al. (2004) were one of the first groups to examine eye gaze in movement imagery; specifically whether imagination of body rotation could induce oculomotor activity similar to the typical vestibulo-ocular reflex. Their data suggested that nystagmic activity in the horizontal plane could be elicited during movement imagery providing evidence that eye movements could be used as an objective measure of online cognitive processes. More recent research has confirmed the significant role of eye movements in movement imagery with further evidence for functional congruence of eye movements between the covert and overt states (Heremans et al., 2008, 2011; McCormick et al., 2012, 2013). These studies are discussed later in this paper.

## Congruency of gaze metrics

Recording eye movements provides an unobtrusive, sensitive, real-time behavioral index of on-going visual and cognitive processing (Liversedge and Findlay, 2000). This indirect, objective experimental approach has been used successfully to compare behaviors between the observation and imagery by a number of research groups (Flanagan and Johansson, 2003; Heremans et al., 2009; McCormick et al., 2012). Collectively, the findings suggest there are similarities but also some discrete differences between the gaze metrics. The following section provides an overview of this literature and is organized by the states compared.

### Action execution and action observation

Flanagan and Johansson (2003) showed that the eye movements of participants observing actors who were performing a block-stacking task were spatially similar to, and in phase with, the eye movements they produced when they performed the task themselves. In both instances, attention was directed proactively to the upcoming point of contact, anticipating the outcome of the actions without attending to the visual unfolding. These anticipatory eye movements in reaching tasks have also been demonstrated in infants as young as 14 months old (Gredebäck et al., 2009). Rotman et al. (2006), in a follow-up study to Flanagan and Johansson, examined eye movements of predictable and unpredictable actions during a similar block-stacking task. Participants observed an actor picking up one of two blocks. The results showed that the observers were able to fixate the goal (target block) through proactive gaze in advance of the actor's hand reaching the goal. These studies suggest that observers are activating their own movement representations for the task being performed by the actor and provide support for the direct matching hypothesis. It should be noted that not all studies have demonstrated proactive eye movements during reach and grasp action observation conditions (see Gesierich et al., 2008), this could be a direct consequence of the instructions provided to participants.

Ambrosini et al. (2011) examined whether these representation transferred into more complex scenarios, where more objects of varying shapes and sizes are present, and whether participants could predict the target object. In a similar set-up, an actor reached for one of two objects, which require two different types of grip. In a control condition the actor did not pre-shape their hand, in the experimental condition the hand was pre-shaped depending on the target object. The results showed that, in the pre-shaping condition, observer's demonstrated earlier saccadic eye movements and higher hand position accuracy compared to the control condition. These data suggest that simply pre-shaping the hand is enough for an observer to identify a target object, providing further support for the idea that when observing others we access the same motor representations as action execution.

Building on these ideas, Ambrosini et al. (2012) asked participants to observe an actor reaching for a target object whilst their hand was either free to move or restrained. Gaze behavior was significantly compromised in the restrained condition with the authors concluding that, when observing actions, it is critical for the observer to be under the same constraints as in action execution. This concept is supported by Costantini et al. (2012), who found that when observing an actor reaching for a target object that was out of reach, proactivity of the observers gaze was compromised.

For relatively simple movements, observers pick up invariant spatial and temporal features from the modeled actions (Mataric and Pomplum, 1998). For instance, in the observation of upper limb movements involving no target, individuals typically fixate the hand or end point, regardless of whether or not the whole limb is used. In situations where the immediate target is unknown, or ambiguous, the observer makes use of other salient motor cues, such as hand pre-shaping, to help identify the appropriate target (Maslovat et al., 2010; Ambrosini et al., 2011). In a similar manner to assisted imagery (Holmes and Collins, 2001), these data suggest information rich visual cues can facilitate observation with the remaining movement details “filled in” using internal models of limb kinematics.

In observation that includes an agent explicitly, the observer's eye movements frequently follow a characteristic sequence. Specifically, the observer typically fixates the agent (generally the agent's head) and then the target (Webb et al., 2010; Letesson and Edwards, 2012; McCormick et al., 2012). It could be that the agent's gaze (and hand trajectory) provides early cues about the anticipated target or goal of the action and/or the sequence is a consequence of specialized neural networks involved in action perception. If action observation uses the same sensorimotor mechanisms as is involved in executing actions, then perhaps observers first attend to the agent to engage these mechanisms or as a necessary pre-requisite of anticipating the target.

A major difficulty in learning through observation is that although individuals are presented with a “model” comprising the task relevant actions, anatomical relationships, kinematic parameters and relative timings, learners may not attend to the important visual information cues. This may occur as a result of divided attention, or because the critical visual cues are not subjectively deemed as “informative” by the observer (Loftus and Mackworth, 1978), detrimentally affecting subsequent performance (Fernandes and Moscovitch, 2000). In addition, a direct relationship between action execution and action observation implies bi-directionality (Schutz-Bosbach and Prinz, 2007); if perceiving action leads to activation in motor areas then action production (by the self) should also prime action perception. In this regard, if action (by the self) is ineffective (e.g., in movement dysfunction after stroke), then this may influence the patient's perceptual sensitivity to the actions of others. Indeed, Underwood et al. (2009) have demonstrated that domain expertise (enhanced top down knowledge) influences gaze both at recognition and memory recall. Experts in different domains demonstrated more consistent scan patterns when viewing domain specific images, compared to images from an unfamiliar domain. That said, researchers have reported that patients who used action observation as part of their stroke rehabilitation therapy (Ertelt et al., 2007) were able to demonstrate physical improvements compared to controls. These data suggest that during action observation we take advantage of the same motor knowledge that enables us to perform actions. In this regard, the action processing might be modulated by our own motor repertoire as well as the importance we attach to the visuomotor information. In situations where the latter two variables are less than optimal, (re)training effective gaze may improve the level of proficiency achieved through this covert approach to motor (re)learning.

### Action execution and movement imagery

Heremans et al. (2008) were the first to compare eye movements between physical execution and subsequent movement imagery. Using a cyclical aiming task the authors reported that 89% of participants made task-related eye movements during imagery with the eyes open and 84% did so during imagery with eyes closed. Furthermore, both the number and amplitude of the eye movements during imagery resembled closely those of eye movements made during the physical execution of the task. The findings contrast, in part, those of McCormick et al. (2013) who reported that additional fixations that were made during physical execution. The differences may be explained through the demands of the actions performed. Heremans et al. (2008) used a relatively low demand cyclic wrist extension action that was cued externally whereas McCormick et al. (2013) employed a task that involved the optimal movement of a stylus to a target in the sagittal plane. These data suggest that the neural coupling that exists between the eye and hand movements during physically executed movements remains partially intact in imagery (i.e., fixation location is preserved). However, the difference in the baseline level of task demand appeared to be uninterpreted in imagery.

In attempts to elucidate the role of eye movements during movement imagery, some researchers have employed chronometry paradigms and included conditions in which eye movements are fixed or free (Gueugneau et al., 2008; Debarnot et al., 2011). Using a joystick tracking task, under normal and mirror conditions, Debarnot et al. (2011) reported that performance accuracy and temporal similarity between physical execution and movement imagery is maintained in the normal condition for eyes-free and eyes-fixed, which suggests that eye movements perform no functional role. However, in the mirror condition the temporal congruence between action execution and movement imagery was maintained only in the eyes-free condition. These data could have occurred as a result of participants in the eyes-fixed condition fixating a cross positioned mid-way between the targets suggesting that peripheral vision may have been used and, given the comparable levels of performance in the normal condition, assisted the task. In more complex tasks, the use of peripheral, rather than high acuity foveal vision may compromise accuracy and results in reduced task proficiency.

In a training study, Heremans et al. (2011) used a Virtual Radial Fitts task where participants were required to have eyes-fixed or allowed spontaneous eye movements. They moved a pen to several targets using their dominant and non-dominant hand. Both groups received movement imagery training; the eyes-fixed group was asked to fixate a red target during the training, whereas the eyes-free group had no eye movement instructions. Results showed that eye movements during movement imagery did not affect the temporal parameters of the action, such as movement time and time to peak velocity, but assisted movement accuracy. These effects were most pronounced in the conditions with high accuracy demands. Effects were found for both the dominant and non-dominant body side, indicating that the effects of movement imagery practice and the role of eye movements during movement imagery practice may be effector- independent.

These studies imply that some of the functional eye movements involved in planning (i.e., determination of the target in the visuomotor workspace) are performed similarly in action execution and movement imagery. It appears that some temporal aspects of gaze (e.g., the functions involved in the online correction of physical movement) are not replicated in imagery.

### Action observation and movement imagery

The concept of a motor representation which is shared by all three simulation states suggests that some gaze metrics should be congruent between action observation and movement imagery, in the absence of any priming action execution. To test this idea, McCormick et al. (2012) used a reach-grasp-place task to examine the gaze congruency between these two conditions and also manipulated visual perspective (first and third person). In the action observation condition participants were instructed to observe the action with the intention to imitate it at a later time. The data showed that although the total number of fixations between conditions (action observation and movement imagery) and perspective was not significantly different, the number of fixations to specific regions of interest (grasp and placement sites) was significantly greater in first, compared to third person perspective. These data suggest that the task related spatial information is influenced by visual perspective; in the absence of a third party agent, information is primarily gathered from the object stimuli. Similar findings have been reported by other research groups (e.g., Letesson and Edwards, 2012). In contrast, McCormick et al. (2012) fixation duration was reported to be significantly longer in action observation than movement imagery. Based on the findings of Loftus and Mackworth (1978), it is suggested that the increased fixation duration reflected the information rich environment of action observation and associated increase in cognitive demand. The number of fixations to target stimuli appears reduced in action observation, and any subsequent movement imagery, when the agent's gaze is visible. Although Humphrey and Underwood (2010) report that the inclusion of social information during picture viewing improves recognition accuracy, it is unknown if social gaze is interpreted in movement imagery and whether it benefits (re)learning in a similar way to action observation.

### Action execution, action observation, and movement imagery

To date, only one study has compared gaze metrics in all three states within a single paradigm. McCormick et al. (2013) conducted a tri-state comparison of the fixation metrics using a forward reach and point Virtual Radial Fitts' Task. The task required participants to reach and point to three different sized targets on a touchscreen with a stylus. The imagery task was executed in the first person perspective with visual cues (guided imagery) and without cues (unguided imagery). As a manipulation check, simulated movement time during imagery was also recorded. Participants fixated the target in all conditions indicating that similar visual and/or extra-retinal information was acquired in conditions. In contrast to the findings of others (Heremans et al., 2009), more fixations were made to the target during action execution but, in support of McCormick et al. (2012), the number of fixations were comparable between action observation and imagery. The increase in the number of fixations during action execution suggests that corrective fixations occurred during the “homing in” phase of the movement (Elliott et al., 2001). This process of guiding the effector using visual feedback is absent in the covert states. Fixation duration was congruent between action execution and action observation; in both conditions the fixation duration increased as task complexity increased. This increase in fixation may be due to the additional online information processing required in the more complex tasks (Brouwer et al., 2009), due to the eyes remaining fixated at the target until the imminent arrival of the limb (Gowen and Miall, 2006), or a combination of both. In either scenario, the fixation duration in action observation appears to mirror that of action execution and suggests the motor representation, inclusive of eye movements, is shared in these states (Flanagan and Johansson, 2003). The authors also reported that movement time was longer in the imagery conditions compared to action execution and, in contrast to fixation duration, the movement times were constrained by Fitts' Law (Fitts, 1954). As fixation duration remained constant during imagery, and the number of target fixations was comparable with action observation, the authors reasoned that information was attended to differently during imagery and that no online corrective functions were simulated. The inter-state differences and similarities uncovered through these direct tests of the simulation theory highlight that the neural sharedness is partial and differentiated by state. Tri-state comparison therefore, permits identification of the specific gaze characteristics that are congruent between states and guides the further optimization based on a neural sharedness model (Jeannerod, 1994) and this information should be exploited to optimize the effectiveness of observation and imagery interventions.

## Implications

We have identified that there are several gaze metrics (e.g., fixation duration and frequency) that have been demonstrated to be congruent between action and simulation states. We have also demonstrated however, that there are several gaze metrics that differ between states. We therefore, encourage practitioners, clinicians, and researchers to consider eye movements and gaze metrics when developing training interventions and therapies, but to be aware that not all gaze metrics are congruent. When designing observation and imagery programs, critical eye movements that are relevant for the given action need to be considered. These important metrics will depend on the task, the context and the individual differences of clients and patients; age, experience, and ability for example.

Practitioners employing imagery and action observation techniques need to be aware not only of the central and peripheral markers, such as cardiovascular responses, but also congruent eye movements as these will provide further evidence that the patients is engaging with the therapy. The transfer of these eye gaze metrics between limbs and similar tasks is also an area of interest and has potential implications for clinical rehabilitation.

### Training tools

The research presented highlights the potential of using action observation and movement imagery to (re)learn or improve skills when physical practice is not an option, or in conjunction with physical practice to optimize motor learning. There is an opportunity to use the data presented above to develop a comprehensive action simulation training or therapy program in a clinical environment. Using a multi- and interdisciplinary approach informed by research in neuroscience and psychology as well as the practice of clinicians, the therapy could support motor leaning or regeneration and neural plasticity through a combination of physical practice, action observation and movement imagery (for reviews see Sharma et al., 2006; Holmes and Calmels, 2008; Garrison et al., 2010). The training or therapy, depending on the client group, would need to bring together concepts of motor planning, action prediction, visual attention, and optimal learning to deliver a personalized action simulation package that simulates motor learning in meaningful and contextually-relevant scenarios.

### Future directions

The majority of the literature presented in this paper has focused on relatively simple tasks requiring limited visual attention. If the ideas and concepts developed from this work are to be translated into real-world domains for use in skill acquisition and rehabilitation, then these concepts need to be examined in more complex environments under a variety of conditions. For example, future research should manipulate task complexity in order to determine when certain gaze variables, such as fixation duration of saccade amplitude, “break-down” in each of the simulation states. This may provide information to researchers and practitioners looking to train skills using the three different states, for example, fixating a target location for longer and/or earlier (Causer et al., 2011).

Despite the growing research interest in action observation and imagery, most of the studies focus on simple tasks using one limb. Many of the actions we perform in daily life involve the simultaneous action and coordination of at least two limbs. Researchers have shown performance limitations during bimanual movements, evidenced through problems in the planning or execution of the independent movements with both hands concurrently (Punt et al., 2005). Asymmetric movements, with different spatial constraints for the left and right hand can also lead to prolonged latencies, distorted trajectories, and high error rates. These factors are further complicated when one considers the site of infarction and hand dominance in stroke patients. In unimanual reaching, visual attention is deployed to the target well in advance of movement termination. In bimanual reaching, it has been suggested that the independent movement goals (objects) are attended in to in a serial way in the latter part of the task to correct for movement trajectory error (Riek et al., 2003). In contrast, during movement preparation visual attention is suggested to be simultaneously deployed to the independent goals, but with more attention allocated to goals that are perceived as more difficult (Baldauf and Deubel, 2008). How the independent goals are attended to during movement preparation and throughout simulation in action observation and movement imagery is currently unknown. Researchers should investigate how gaze may be affected and controlled during more complex movements and how these translate onto activities of daily living.

In terms of clinical research directions, more research is needed into the use of gaze metrics in rehabilitation and how to optimize skill (re)learning and increasing movement function. In line with this, identification of video feedback and highlighting task relevant cues, via gaze metrics such as fixation zones and fixation duration, especially in high-risk everyday activities, could potentially reduce accidents and injuries, as well as enable patients to relearn skills more effectively following stroke or other movement dysfunctions.

## Conclusions

In this paper we have reviewed critically the literature on eye movements in action execution, action observation, and movement imagery. We identified gaze variables that are congruent and incongruent across states providing an argument for gaze congruency as an implement for developing action observation and movement imagery interventions. We also identified research that supports the idea of a partially shared neural network between the states. We encourage researchers and practitioners to utilize eye movement metrics in experimental and rehabilitation contexts that are representative of the action execution scenario when using action observation and movement imagery interventions. These guidelines can help us move toward more effective training and skill learning in multiple domains, from high performance sports to clinical rehabilitation.

### Conflict of interest statement

The authors declare that the research was conducted in the absence of any commercial or financial relationships that could be construed as a potential conflict of interest.
